# Thinking Outside of the Box

**DOI:** 10.5812/traumamon.4056

**Published:** 2012-05-26

**Authors:** Mohammad Hosein Kalantar Motamedi

**Affiliations:** 1 Trauma Research Center, Baqiyatallah University of Medical Sciences, Tehran, IR Iran

Advancements in the fields of traumatology and medicine are made based on sound research and scientific methodology. Today, those with novel ideas and innovations pave the path towards better patient healthcare. This in turn translates to lesser morbidity and mortality, shorter hospital stay and less lost work days for the patient. In order for scientists and clinicians to be creative they must be able to keep an open mind and be able to contemplate and concentrate on their work. They must endeavor patiently and persevere relentlessly. Many renowned pioneers of modern medicine such as **Ramon Cajal, Marie Curie** and **Louis Pasteur** among others had such qualities; they patiently pursued a flawless methodology which eventually led to success (1-3). Although their work was tedious, and in essence, trial and error, the aforementioned characteristics were the elements that led to fruitful research and new discoveries. In order for a scientist’s work to reach fruition there must exist an open atmosphere, a calm setting for contemplation, a lab for experimentation, a motivation for research and a place for relaxation. The researcher must be free to explore methods and techniques outside the textbook routine. Moreover, research is costly and thus requires financial support.

Although thinking outside of the box and modifying and/or changing the established academic routine may not be a problem in the basic sciences, however, in the fields of clinical medicine or life sciences this is often not the case. Clinical research requires adherence to international laws, rules and regulations relative to medical ethics, human rights and informed patient consent. Clinicians cannot stray from current treatment guidelines in their practice despite the patients’ consent to undergo experimentation. New drugs, instruments, treatment modals and techniques cannot be implemented until they have been thoroughly assessed and backed by in vivo and in vitro animal studies and final FDA approval. These are standard prerequisites for coining a new technique, new treatment modality or a wonder drug.

Keeping an open mind entices creativity; new concepts and research; ideas may then come right out-of-the-blue. Numerous scientists had undoubtedly seen a ripened apple fall from a tree before **Sir Isaac Newton** (1642 –1727) did (4); however it was he the English physicist, mathematician, astronomer, natural philosopher, alchemist, and theologian, who sought the cause and later coined the laws of gravity. The famous French surgeon **René Le Fort** (1869-1951), derived his classic classification of facial fracture patterns by observing fractures arising from forceful blows subjected to the facial skeleton (5). Hugo Obwegeser an Austrian oral and maxillofacial surgeon invented and introduced to the surgical world, the bilateral sagittal split ramus osteotomy (SSRO) by observing this type of fracture occurring in patients with mandibular fractures; today, SSRO is the single most commonly used procedure in orthognathic surgery. A great number of surgical instruments were also devised by surgeons bending or modifying standard instruments. **Myron Firth Metzenbaum** (1876-1944) (6), an American surgeon who specialized in oral and reconstructive surgery designed surgical scissors with tungsten carbide blades for cutting delicate tissues because he noted that ordinary scissors would not cut tissues effectively. **Alfred Washington Adson** (1887–1951) (7), an American doctor was another innovator who developed a traumatic tissue forceps to grasp soft tissues. **Howard Atwood Kelly** (8), professor of obstetrics and gynecology at Johns Hopkins developed a versatile clamp that may be used for occluding blood vessels, manipulating tissues, grasping bone segments for reducing fractures and a variety of other purposes. These examples are just a few of the numerous pioneers who changed the way things were done because they had the insight and dedication their work demanded and their patients required. They did not limit their work to repetition of the normal routine or show apathy towards change but rather sought to fulfill shortcomings, prevent procedural problems and improve their work by refining techniques and thereby enhancing the healthcare rendered. As one can see from the history of modern medicine, innovation is not only the result of perseverance in research, but also keeping an unconfined and open mind; constant contemplation on what we do, how we do it and how we can improve it to benefit mankind (while respecting the ideals, rules and ethical regulations) are key elements for success and scientific progress.
